# Terminal Investment: Individual Reproduction of Ant Queens Increases with Age

**DOI:** 10.1371/journal.pone.0035201

**Published:** 2012-04-11

**Authors:** Jürgen Heinze, Alexandra Schrempf

**Affiliations:** Biologie I, Universität Regensburg, Regensburg, Germany; Université Paris 13, France

## Abstract

The pattern of age-specific fecundity is a key component of the life history of organisms and shapes their ecology and evolution. In numerous animals, including humans, reproductive performance decreases with age. Here, we demonstrate that some social insect queens exhibit the opposite pattern. Egg laying rates of *Cardiocondyla obscurior* ant queens increased with age until death, even when the number of workers caring for them was kept constant. *Cardiocondyla*, and probably also other ants, therefore resemble the few select organisms with similar age-specific reproductive investment, such as corals, sturgeons, or box turtles (e.g., [1]), but they differ in being more short-lived and lacking individual, though not social, indeterminate growth. Furthermore, in contrast to most other organisms, in which average life span declines with increasing reproductive effort, queens with high egg laying rates survived as long as less fecund queens.

## Introduction

According to life history theory, organisms can, in principle, maximize lifetime reproductive success by increasing their reproductive investment with age, as their future reproductive success becomes smaller with approaching death (“terminal investment”, [2], [3]). However, this increase is often obscured by the progressive deterioration of an individual’s reproductive performance with age, which may lead to decreasing reproduction later in life (“reproductive senescence”, e.g., [4]–[6]). Indeed, in numerous long-lived vertebrates, including humans, and also many short-lived invertebrates, reproductive investment strongly declines with age (e.g., [7]–[10]).

Though the extraordinarily long life span and fecundity of social insect queens has recently attracted the attention of researchers interested in the association between life span and reproduction, little is known on the age trajectory of reproduction. Queens in particular of ants and termites are well protected from external hazard by their workers and by far outlive similarly-sized non-social insects and also their own workers. This matches the association between aging and extrinsic mortality predicted by evolutionary theories of aging [11]. In addition, the special life history of queens, which first build up a colony consisting of non-fertile workers and switch to the production of sexuals only later in life, suggests that the strength of selection may remain high even at older age [12]. Mutations that negatively affect the fecundity of queens late in life will be selected against. From this we expect that the reproductive effort of ant queens does not decrease dramatically with age. To test our prediction, we monitored life spans and weekly egg laying rates of 25 *Cardiocondyla obscurior* ant queens throughout their complete lives. *C. obscurior* is particularly well suited for such a study because its colonies are very small (∼20 –30 workers and 1 to 5 queens [13]) and its queens are relatively short-lived (mean life span 26 weeks [14]). The lifetime brood production of queens therefore can be monitored relatively easily.

## Methods

All animal treatment guidelines applicable to ants under international and German law have been followed. Collecting the colonies that form the basis of the laboratory population used in this study was permitted by the Brazilian Ministry of Science and Technology (RMX 004/02). No other permits were required for this study.

*Cardiocondyla obscurior* (Wheeler, 1929) is a small ant, both concerning individual body size (∼ 2 mm) and colony size (see above). Queens mate in their natal nests and later may leave with a few workers and brood to establish their own nests nearby. We set up 25 experimental colonies from a large laboratory population established from nests originally collected in an experimental plantation in Una, Bahia, Brazil. Female sexuals were allowed to mate with a single, wingless male each and placed into standard plastic nest boxes with 20 workers from their maternal colony. Colonies were housed under near-natural conditions (e.g., [14] in incubators (12 h 30°C/12 h 25°C) and fed twice per week with diluted honey and chopped up cockroaches ad libitum. Eggs were counted once per week and the number of adult individuals in the colony was standardized to 20 workers per colony between colonies by removal of all pupae. In this way, we attempted to keep the costs of reproduction constant. As it takes approximately one week for an egg to develop into the first larval instar [15] and workers are incapable of laying eggs, weekly egg counts give a reliable estimate of the queen’s actual egg laying activity. While queen age may affect embryo mortality in the more long-lived queens of honeybees [16], we do not have any indication that egg hatching rates differ with the age of queens (unpublished observations).

Because of the small size and weight of queens (dry weight 5.7 × 10^−5^ g, [17]), changes in queen body mass could not be determined. However, adult ant queens do not grow and changes in weight are usually associated with the swelling of the abdomen and thus reproductive status (e.g., [18]). This means that a queen’s energy allocation to individual maintenance and survival more or less remains constant and egg laying rate reflects allocation to the production of offspring.

As data were not normally distributed we applied non-parametric statistics using Statistica 6.0. Because many of the measured parameters were interrelated we chose to analyze the associations with several individual Spearman rank correlation tests with sequential Bonferroni correction where appropriate.

## Results

**Figure 1:**
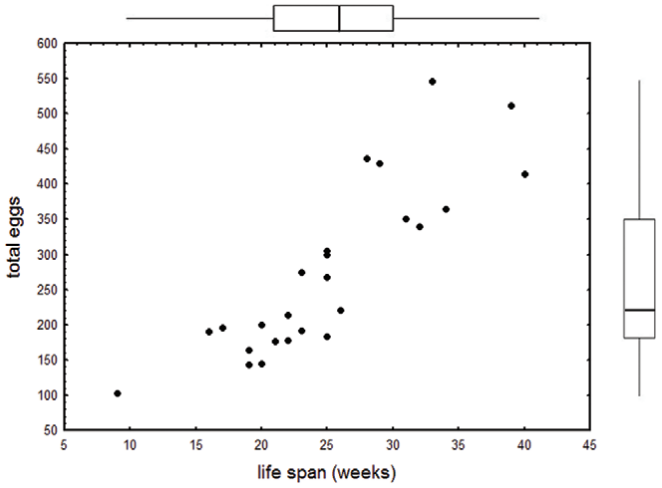
Association between life span and the total number of eggs laid by queens of the ant *Cardiocondyla obscurior*. Bars and whiskers indicate median, quartiles and range of egg number (right) and life span (top).

The summed weekly egg counts (median 220, quartiles 183, 350) were positively associated with life span, i.e., queens that laid more eggs also lived considerably longer (r_S_  =  0.873, p < 0.0001). This is in part a simple consequence of longer lived queens being capable of laying eggs over a longer period of time. Life span explained approximately ¾ of the variance in total egg number (linear regression total eggs vs. life span β  =  0.858, p < 0.0001, r^2^  =  0.736, Fig. 1), but was also weakly, but not significantly associated with the mean number of eggs present per week (r_S_  =  0.339, p  =  0.098).

**Figure 2:**
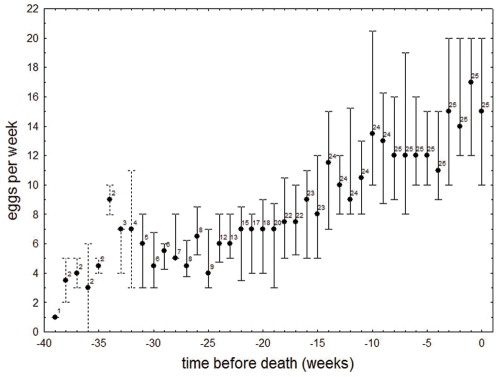
Association between time to death and the weekly egg count in colonies of the ant *Cardiocondyla obscurior*. Sample sizes are given by the numbers near median. Whiskers indicate median and quartiles for sample sizes larger than four colonies and range for sample sizes of 3 and less.

Egg laying activity increased with age, as documented by a significantly positive correlation between weekly egg count and week of life in 21 of 25 colonies (r_S_ between 0.555 and 0.949, p-values after Holm’s Bonferroni correction < 0.002 in 19 colonies and < 0.02 in two colonies). As life span varied considerably among individual queens (median 25 weeks, quartiles 29, 20 weeks), we chose to illustrate this association by plotting egg laying rates over time before the queens’ death. Fig. 2 shows that egg laying rates increase with queens approaching the end of their lives. The mean number of eggs laid per week was significantly lower than the mean number of eggs laid during the last two weeks of a queen’s life, even when data from the first two weeks were excluded (sign test, n  =  25, Z  =  4.000, p < 0.0001). Egg number was slightly, but insignificantly lower in the last week than in the second to last week of the queen’s life, i.e., queens were still highly fecund a few days before they died (median 17 vs. 15, sign test, n  =  25, Z  =  0.204, p  =  0.838). Our data reveal neither a negative impact of early reproduction (eggs laid per week during the first quarter of the life time) on life expectancy (r_S_  =  0.066, p  =  0.752) nor a trade-off between early and late reproduction (eggs laid per week during last quarter of life time; r_S_  =  0.244, p  =  0.239).

## Discussion

Our study yields two interesting results. First, the sum of weekly egg counts increased with queen life span. In social Hymenoptera, eggs may develop into sterile workers instead of female and male reproductives, and egg counts are thus not an infallible one-to-one measure of reproductive success. Genetic variation in the propensity of diploid eggs to develop into workers or young queens [19], [20] may obscure the association between total eggs laid and reproductive success. The fate of eggs has not been examined in our study. However, re-analysis of an earlier data set, in which worker number per colony was not kept constant [21], shows that the total number of sexuals produced by queens mated with wingless males is strongly positively correlated with life span (n  =  50, total number of sexuals produced, r_S_  =  0.591, p < 0.0001; female sexuals, r_S_  =  0.499, p < 0.0003; winged males, r_S_  =  0.509, p < 0.0002; wingless males, r_S_  =  0.568, p < 0.0001). Assuming that embryo mortality does not change drastically with queen age (but see [16]), it is therefore likely that higher queen productivity would also have resulted in a higher number of sexuals in our study if eggs had been allowed to develop.

Beyond that, colony size was kept constant in the present study, and as increasing worker number presumably positively affects total productivity [22], [23] the effect of queen life span on reproductive success might be even more pronounced under natural conditions. Our result therefore parallels findings in bumble-bees, where a queen’s lifetime reproductive success also increases with its longevity [24]. Both studies match the well-known observation that, in contrast to many other animals, individual reproductive effort is not traded off against individual life span in the social Hymenoptera. For example, randomly chosen workers of the parthenogenetic ant *Platythyrea punctata*, which were allowed to reproduce, outlived their non-reproductive clone-mates [25], and both mated and unmated egg-laying workers of the queenless ant *Diacamma* sp. survived significantly longer than unmated, non-reproductive workers [26], [27]. In *C. obscurior*, virgin queens, which have a low egg laying rate, reached only about 2/3 of the life span of mated, fertile queens [14], and the completely sterile workers live for an average of 15 weeks (Marcel Medrano, personal communication). In social insects, the life-shortening “costs of reproduction” [28] are presumably not born completely by the reproductive individual alone but compensated through the activities of the whole society (e.g., [29]).

Second, the egg laying rate of queens of *C. obscurior* increased with age, and the number of eggs queens laid during the last two weeks of their lives was usually significantly higher than the average number of eggs laid per week. The very low egg laying rates during the first weeks after mating may reflect the limitations of a not yet fully active reproductive system. Queens of ant species that engage in highly synchronized nuptial flights and found new nests solitarily may have their first eggs ready for fertilization immediately after mating (unpublished results from dissections of queens of *Lasius niger* and other species caught after a nuptial flight). In *C. obscurior*, where young queens may stay and eventually mate in their natal nests, the ovaries of young virgin queens do not contain maturing, yolky eggs (unpublished results). However, these initial constraints do not explain the steady increase of egg number several months after mating. Even though we cannot completely exclude a decline of fecundity immediately before death, our observations therefore do not give evidence for “reproductive senescence.” This stands in contrast to the common finding that the fecundity of insect females decreases at late age (e.g., in *Drosophila*: [7], [30]; pentatomid bugs: [31]; beetles: [8], [32]; parasitoid wasps: [33], [34]; crickets [35]).

Given that worker number – and therefore the provisioning of the queen - was kept constant we conclude that in *C. obscurior* colonies, the reproductive investment of queens, i.e., the proportion of resources committed to the production of offspring [36], increases throughout their lives and reaches a maximum shortly before death. We did not directly quantify reproductive allocation, i.e., the distribution of resources to worker vs. sexual offspring. However, it is likely that reproductive allocation will also increase with age, because after the death of the queen workers rear mostly female sexuals from its brood [37]. *C. obscurior* thus resembles the few organisms, in which an increase of reproductive efforts with age has been demonstrated. This includes organisms with modular growth, such as corals or plants that develop in clonal clusters, or organisms with indeterminate growth, such as many fish and reptiles, in which fertility increases with body size [1]. Individual adult queens of *C. obscurior* do not increase in size, but their colonies do. Employing the concept of the “superorganism,” in which queens are regarded as the “reproductive organs” of steadily and modularly growing insect societies [38], the reproductive schedule of ant queens becomes an interesting analogy to those of solitary organisms with life-long growth.

The age trajectory of reproduction in *C. obscurior* might reflect terminal investment [2], [3] or negative reproductive senescence, i.e., a decline of mortality with age, accompanied by an increase in fecundity [1]. Unfortunately, our sample size is too small for the determination of age-specific mortality rates to distinguish between these two hypotheses. However, mortality presumably does not increase strongly with age, as queens are shielded by workers from external risks [11]. Future studies will show how the observed pattern responds to colony growth and the adoption of young queens. The collective productivity of queens in multi-queen colonies of the little fire ant, *Wasmannia auropunctata*, reached a maximum at middle age and later decreased again [39], suggesting that under these conditions queen fecundity may to some extent decline at old age.
